# AM fungi patchiness and the clonal growth of *Glechoma hederacea* in heterogeneous environments

**DOI:** 10.1038/srep37852

**Published:** 2016-11-25

**Authors:** Nathan Vannier, Anne-Kristel Bittebiere, Philippe Vandenkoornhuyse, Cendrine Mony

**Affiliations:** 1 Université de Rennes 1, CNRS, UMR 6553 EcoBio, Campus Beaulieu, Avenue du Général Leclerc, 35042 RENNES Cedex (France); 2 Université de Lyon 1, CNRS, UMR 5023 LEHNA 43 Boulevard du 11 Novembre 1918, 69622 VILLEURBANNE Cedex (France)

## Abstract

The effect of AM fungi spatial distribution on individual plant development may determine the dynamics of the whole plant community. We investigated whether clonal plants display, like for other resources, a foraging or a specialization response, to adapt to the distribution of AM fungi. Two separate experiments were done to investigate the response of *Glechoma hederacea* to a heterogeneous distribution of a mixture of 3 AM fungi species, and the effects of each species on colonization and allocation traits. No specialization and a limited foraging response to the heterogeneous distribution of AM fungi was observed. An effect of the AM fungal species on plant mass allocation and ramet production, but not on spacer length, was detected. Two possible explanations are proposed: (i) the plant’s responses are buffered by differences in individual effects of the fungal species or their root colonization intensity. (ii) the initial heterogeneous distribution of AM fungi is perceived as homogeneous by the plant either by reduced physiological integration or due to the transfer of AM fungi propagules through the stolons. Microscopic and DNA sequencing analyses provided evidence of this transfer, thus demonstrating the role of stolons as dispersal vectors of AM fungi within the plant clonal network.

In nature, environmental conditions, especially resources, vary spatially and temporally even at a fine scale. The spatial variations in resources abundance are perceived by organisms as environmental heterogeneity so long as the patches of resources are smaller than the organism and larger than the response unit^1^,^2^. Plants, because of their sessile lifestyle, have to cope with this heterogeneity and have evolved complex and diverse buffering mechanisms, such as phenotypic plasticity (*i.e.* production of different phenotypes from a single genotype^3^). Phenotypic plasticity improves the plant’s ability to respond to resource heterogeneity during its lifetime by allowing trait adjustment to current environmental conditions^4^,^5^,^6^,^7^. Plasticity is expressed at different modular levels in plants^8^, ranging from first order modules such as leaf or root to a higher modular level such as the ramet (see Harper, 1977 for modular structure description^9^). This plastic response results from a trade-off between environment exploration for a resource (*e.g.* foraging for nutrient-rich patches) and resources exploitation (*e.g.* uptake of the resource and establishment within the patches).

In clonal plants, each individual consists of a set of ramets connected through belowground (*i.e.* rhizomes) or aboveground horizontal modified stems (*i.e.* stolons). These connections result in a network structure and promote plant propagation in space (*i.e.* physical integration). In some species they also allow sharing of information and resources within the physical clone (*i.e.* physiological integration^10^). As a result of this network architecture, clonal individuals experience spatial heterogeneity at centimetric scales. They also share information about this environmental signaling between ramets. This leads to plastic responses at the local scale to optimize performance, through resource-sharing, at the clone level^11^. The response of clonal individuals to this small-scale heterogeneity results from a resource exploitation-exploration trade-off. Exploration responses are mostly linked to ramet positioning and induce modifications in the clonal network architecture to allow foraging for available resources^12^,^13^. The optimal foraging theory predicts that ramets should maximize resource acquisition by aggregating in rich patches and avoiding poor patches^12^,^14^,^15^,^16^. Such aggregation may be achieved through modifications of the horizontal architecture of clonal plants, such as internode shortening or increased branching^12^,^17^,^18^. Exploitative responses involve changes in resource acquisition traits. As a result of physiological integration, each ramet may specialize in acquiring the most abundant resource (division of labor theory^19^) and share it throughout the network. This specialization can involve modifications in ramet resource allocation patterns^20^,^21^ whereby a higher root/shoot ratio is observed in ramets developing in nutrient-rich patches, and a lower ratio in light-rich patches^20^,^22^.

Clonal foraging and ramet specialization have been demonstrated in response to soil nutrient heterogeneity^22^,^23^,^24^,^25^. However, under natural conditions, plant-nutrients uptake is mostly mediated by symbiotic micro-organisms such as Arbuscular Mycorrhizal (AM) fungi which colonize ~80% of terrestrial plants^26^. AM fungi symbionts (*i.e.* Glomeromycota) colonize roots and develop a dense hyphal network, exploring soil to ‘harvest’ mineral nutrients for the plant’s benefit^26^. Plants with mycorrhized roots can thus attain higher rates of phosphorus and nitrogen absorption (x 5 and x 25 respectively) than plants with non-mycorrhized roots^27^,^28^. In turn, AM fungi obtain from plants the carbohydrates required for their survival and growth^29^,^30^. Under natural conditions, plant roots are colonized by a complex community of AM fungi^31^. These fungi display different levels of cooperation ranging from good mutualists to more selfish ones (*i.e.* cheaters^32^). Within the root-colonizing fungal assemblage, plants have been shown to preferentially allocate carbon to the best cooperators, thereby favoring their maintenance over cheaters^33^. The additional nutrient supply provided by AM fungi can be assimilated as a resource for the plant. (An important raising expectation is that plants may respond to the heterogeneous presence of AM fungi as they do for a nutritive resource. Thus the plant might forage (optimal foraging theory) or specialize (division of labor theory) in response to AM fungi presence. The opposite hypothesis is that AM fungi and foraging or specialization are alternatives to cope with resource heterogeneity, implying that plants with clonal mobility do not rely on AM fungi to respond to this heterogeneity.

Our aim in this study was to analyze a plant’s plastic response to AM fungal heterogeneity by performing two experiments under controlled conditions with the clonal herb *Glechoma hederacea*. In the first experiment, we tested the plant’s foraging and specialization response to the heterogeneous distribution of AM fungi. The treatments consisted of a mixture of three species of AM fungi that had been shown to display various degrees of cooperativeness in precedent studies. Two assumptions were tested: (i) according to the optimal foraging theory, clones should aggregate ramets in the patches containing AM fungi by reducing their internodes lengths and (ii) according to the division of labour theory, clones should specialize in producing ramets with a higher allocation to roots in the presence of AM fungi than in their absence. To better understand the results obtained in experiment 1 and because of the potential impact of different levels of cooperation in the fungi involved in this symbiosis, we carried out a second experiment to test the effect of AM fungal identity on the foraging and specialization response of *G. hederacea*. We tested i) the effect on plant traits of the individual presence of the three different species of AM fungi used in the assemblage treatment and ii) the assumption that AM fungal species differ in their effects on the traits involved in foraging and specialization responses. In both experiments, the performance of clonal individuals was expected to be reduced in the absence of AM fungi.

## Results

*G. hederacea* traits variation was not significantly influenced by plant genotype in either experiment (*i.e.* the inter-genotypic variance was not greater than the intra-genotypic variance).

### Experiment 1: Effect of heterogeneous AM fungi distribution on *G. hederacea* foraging and specialization responses

The hypothesis of modified foraging and specialization responses of *Glechoma hederacea* to the patchiness of AM fungal presence was tested by comparing the internode lengths and R/S ratio between the treatments for the 5^th^, 6^th^, 10^th^ and 11^th^ ramets (see Methods for details on ramet selection and experimental design).

**Figure 1:**
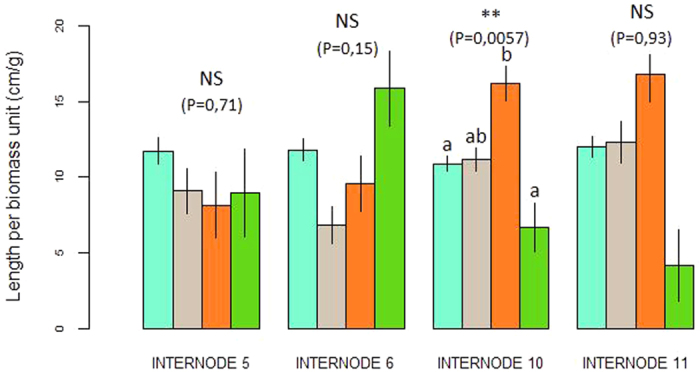
Foraging response: internode length under the four treatments applied (cm per gram of ramet total biomass) (A). Specialization response: root:shoot ratio (R/S) of 5^th^, 6^th^, 10^th^ and 11^th^ ramets under the four applied treatments (g of roots per g of shoots after drying) (B). Absence (blue bars), Presence (grey bars), Presence-Absence (orange bars), Absence-Presence (green bars). Statistical significance of the internode length or R/S variations between treatments: NS, not significant; **P < 0.01.

A significant effect of the AM fungal treatment was found on the 10^th^ internode length (*P* = 0.005; F = 5.74) (Fig. 1) with a longer internode in the PA treatment (AM fungi initially present then absent) than in the absence (A) and presence (P) treatments (results are presented in Table 1). Conversely, no significant effect was found for the 5^th^ ramets (*P* = 0.71; F = 0.45) or 6^th^ ramets (*P* = 0.15; F = 1.92) (Fig. 1). The 11^th^ ramets seemed to display the same response patterns as the 10^th^ ramets, but no significant differences were detected between the treatments (P = 0.93; F = 0.15), due to a partial bimodal distribution of data in the “P” treatment with a few individuals exhibiting longer stolons. In addition, the number of ramifications produced by the 5^th^, 6^th^, 10^th^, and 11^th^ ramets was not significantly affected by treatment. No changes in the R/S ratio in response to AM fungal treatment were detected in any of the four tested ramets.

As regards performance, *G. hederacea* growth rate tended to vary with the AM fungal treatment (*P* = 0.067; F = 2.7), with a tendency for slower growth in the “A” treatment. No differences between treatments were detected for clone total biomass (*P* = 0.75; F = 0.39) which indicated that the clone, as a whole, did not exhibit any difference in biomass production or performance.

### Experiment 2: Effect of AM fungi identity on *G. hederacea* traits

**Figure 2:**
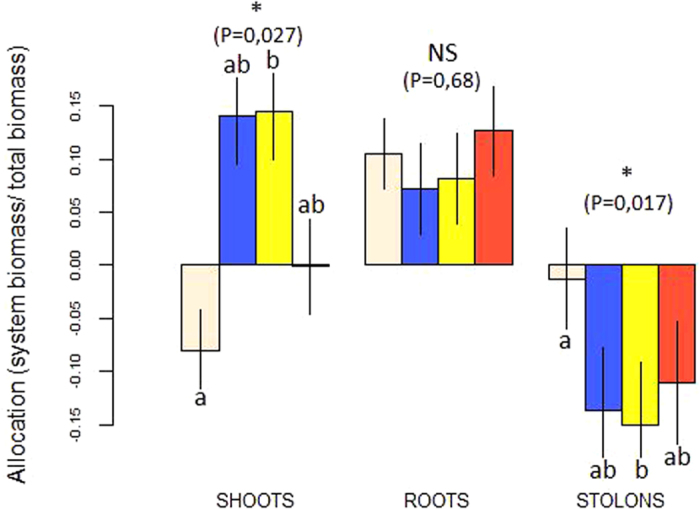
Allocation traits of the whole clone for the four treatment of AM fungi inoculation: T1 = no AM fungi (white bars), T2 = *Glomus custos* (blue bars), T3 = *Glomus intraradices* (yellow bars), T4 = *Glomus clarum* (red bars). Mean biomass of each organ (shoots, roots and stolons) in grams per gram of total clone biomass. Statistical significance of the organ biomass variations between treatments: NS, not significant; *P < 0.05.

The hypothesis that modifications in *G. hederacea* foraging and specialization traits were affected by the AM fungal species was tested by comparing the allocation, architectural and growth traits of four treatments inoculated with different AM fungal species (see Methods for details on experimental design). Primary stolon length (an architectural trait) tended to vary (*P* = 0.07; F = 2.83) in response to the presence and species of AM fungi whereas the number of ramifications (*P* = 0.25; F = 1.49) did not (results are presented in Table 2). Allocation to stolons was significantly affected by the presence and species of AM fungi (*P* = 0.017; F = 4.51) with plants inoculated with *Glomus intraradices* allocating significantly fewer resources to stolons (Fig. 2) and more to shoots (*P* = 0.019, F = 4.24) than plants without AM fungi. The allocation to roots, however, was not dependent on the treatment (*P* = 0.68; F = 0.50).

**Figure 3:**
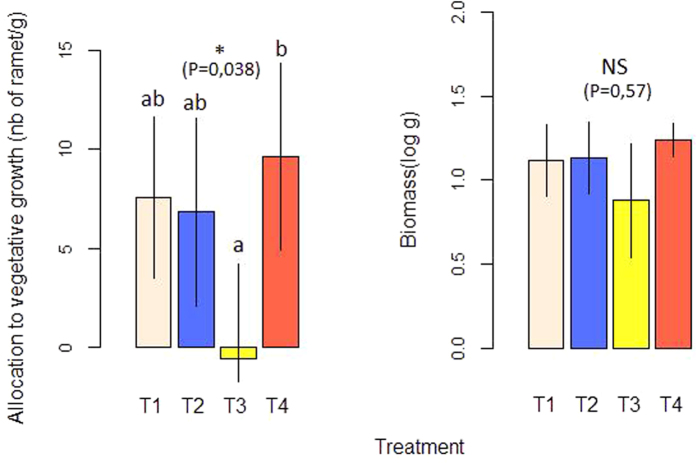
Performance traits of the clone for the four treatments of AM fungi inoculation: T1 = no AM fungi (white bars), T2 = *Glomus custos* (blue bars), T3 = *Glomus intraradices* (yellow bars), T4 = *Glomus clarum* (red bars). Total clone biomass in grams after drying (A). Number of ramets per gram of total clone biomass (B). Statistical significance of the total biomass and number of ramets variations between treatments: NS, not significant; *P < 0.05.

As regards performance, changes in ramet production per biomass unit (P = 0.038; F = 3.55) were detected with *G. intraradices* inducing less ramet production than *G. custos,* whereas the treatments without AM fungi and with *G. clarum* did not differ significantly from the other two treatments (Fig. 3). No treatment-dependent change in total biomass was observed (*P* = 0.57; F = 0.67).

## Discussion

The plants did display some foraging behavior in response to AM fungi heterogeneity, as elongation of the internodes was observed in patches without AM fungi after the plant had experienced patches with AM fungi. This behavior would correspond to an avoidance of resource-poor patches, as expected from the optimal foraging theory. However, this behavior was only detected at a particular ramet age (10^th^ ramets), indicating a possible role of the ontogenic state in development of the plastic response^34^. This may be due to a “lag time” in the plant’s response based on the need for environmental sampling. Indeed, Louâpre *et al.*, (2012) demonstrated that clonal plants may need a minimum number of sampling points as benchmarks in order to perceive and respond to resource availability^35^. In their study, *Potentilla reptans* and *P. anserina* started to respond to the treatment after the 5^th^ internode, suggesting a strong effect of patch size. A similar patch size effect had already been demonstrated in modeling studies^10^,^36^. No plastic modifications, corresponding to a ramet specialization of *G. hederacea* in response to AM fungal spatial heterogeneity, were found either. Contrary to the results expected with the specialization theory, biomass was not preferentially allocated to the roots in patches with AM fungi or to the shoots in patches without AM fungi. This absence of response was recorded for all the ramet ages tested.

These results – a mild foraging response and no specialization – give credit to the theory supported by Onipchenko & Zobel (2000) that species with high mobility do not rely on AM fungi to cope with resource heterogeneity^37^. *Glechoma* with its high clonal mobility should thus show no response to AM fungi presence. However, our results do not fit with the literature predictions for specialization and foraging response^38^. This divergence may be explained by two alternative hypotheses that are developed in the following sections. The first explanation is linked with the occurrence of an individual effect of the species of AM fungus on plant traits, which may predominate or modify the response to the presence/absence of AM fungi when all three species exist together (experiment 2); the second is linked with reduced physiological integration either due to a direct effect of AM fungi on this plant trait, or to the absence of a clear contrast between the different patches sensed by the plant.

In our second experiment, we demonstrated that the architectural traits involved in the plant’s foraging response were not affected by the species of AM fungi tested, which is consistent with the weak response detected in the first experiment. On the contrary, significant changes in resource allocation traits (linked to the specialization response) were detected, depending on the species of AM fungus. Only one species, *G. intraradices* induced a change in allocation by the plant, in comparison to the absence of AM fungi treatment, which led to an increased allocation to shoots at the expense of stolons. Modifications of plant phenotype, depending on the AM fungal species, have already been observed in such traits^39^,^40^. These authors identified a significant effect of *Glomus* species isolates on branching, stolon length and ramet production in *Prunella vulgaris* and *Prunella grandiflora.* In the first analysis of the AM fungal genome, Tisserant *et al.* (2013) revealed existing pathways attributed to the synthesis of phytohormones or analogues^41^. Such molecules would have a direct effect on host phenotype. In the individual effect observed, plant response in the presence of *G. intraradices* symbiosis was coupled with decreased plant performance due to a diminution of ramet production relative to biomass in this treatment. In contrast, the *G. custos* treatment led to a decrease in the potential number of descendants of the clone. According to experiment 1, root colonization by an inoculum containing three species had no effect on plant traits associated with specialization and foraging. This suggests two alternative hypotheses: i) *G. intraradices* may be less cooperative than *G. custos* with *Glechoma hederacea* and the result is a consequence of the plant’s rewarding process to the more cooperative fungus^33^ and/or ii) root colonization by *G. custos or G. clarum* buffers the effect of *G. intraradices* due to a ‘priority effect’ (*i.e.* order of arrival in the colonization as a key to fungal community structure in roots)^41^,^42^.

To test this, the mycorrhization intensity of the three AM fungal species inoculated in the first experiment would need to be assessed by qPCR. Alternatively, the combined effects of the three AM fungal species on plant phenotype might result in the environment not being perceived as heterogeneous by the plant. This hypothesis is developed in the following section.

**Figure 4:**
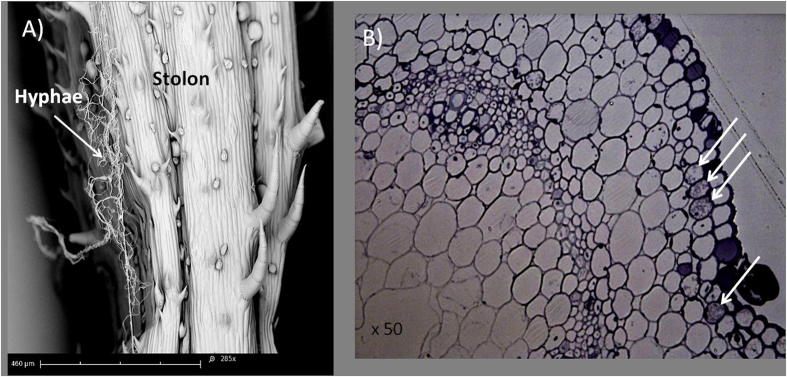
Results for the microscopy analysis of stolons harvested from *G. hederacea* pre-cultures. Scanning electron microscopy of the stolon surface showing hyphae attached to the stolon hairs **(A)**. Stolon microscopy cross-section observed with an optical microscope. Arrows indicate cortical cells invaded by structures which may be interpreted as fungi (**B**).

**Figure 5:**
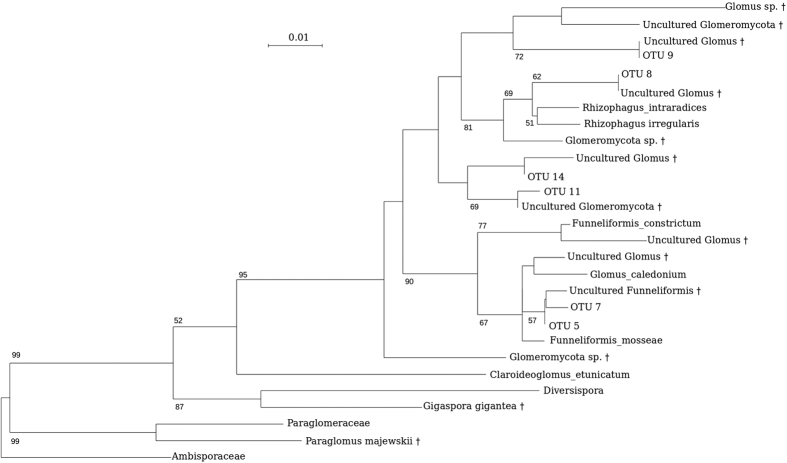
Maximum likelihood tree of the GTR + I + G model using PhyML. Multiple alignment was produced with MUSCLE^62^. Bootstrap values at the nodes were produced from 200 replicates. Only values above 50 are shown. Multiple alignment and tree reconstruction were performed using SEAVIEW^63^. OTUs were obtained from a *Glechoma hederacea* stolon after DNA extraction using the DNEasy plant mini kit (Qiagen), PCR amplification using fungal primers NS22b and SSU817, and Illumina MiSeq sequencing. In addition to reference sequences within the Glomeromycota phylum, we sampled 13 sequences among the best BLAST hits (†).

The intraclonal plasticity predicted by the foraging and division of labor theories is based on the ability of ramets to sense environmental heterogeneity, and to share information and resources within the clonal network, to locally adapt and optimize the performance of the whole clone. The weak response of *G. hederacea* to AM fungal heterogeneity could thus be explained by a decrease in physiological integration that reduces the level of resource-sharing within the clone and prevents the plant from developing an optimized foraging or specialization response. This diminution could initially be due to the presence of AM fungi. Only a few studies have been carried out on the effect of AM fungi on the degree of integration^43^. These authors demonstrated that AM fungi led to reduced physiological integration in the clonal plant *Trifolium repens* when grown in a heterogeneous environment. This effect was dependent on the presence and richness of AM fungal species. Whether this observed diminution of physiological integration would be due to a direct manipulation of the host plant phenotype by the fungi remains, as far as we know, unknown. Secondly, this diminution may depend on the individual plant’s perception of environmental conditions that might be sensed as homogeneous because the patch contrast is smaller than expected. A reduction of plant integration is expected when the maintenance of high physiological integration is more costly than beneficial^44^,^45^, *e.g.* when the environment is resource-rich, not spatially variable^46^ or insufficiently contrasted^10^,^47^. Such a reduced contrast might result from the effect of the three AM fungal species on the plant phenotype (when used as a mixed inoculum), which is unlikely. A more probable mechanism of environment homogenization could result from AM fungal transfer through the stolons. Scanning electron microscopy of the clone cultures (see protocol in supplementary material) revealed the presence of hyphae on the stolon surface (Fig. 4). In addition, several cells close to the external surface of the stolon cross-section were invaded by structures which could be interpreted as fungi. DNA sequencing of stolon samples (Fig. 5) confirmed these results and demonstrated the presence of AM fungi in the stolons. This suggests that fungi can be transferred from one ramet to another, at least by colonization of the stolon surface (as shown in Fig. 4A) and/or within the stolon (Fig. 4B). Whether fungi are passively or actively transferred through the plant’s stolon tissues, and hence to all related ramets, remains an open question. Further studies are therefore needed to confirm these fungal transfers to plant clones and to measure their intensities in contrasted environments.

Studies of the response of clonal plants to environmental heterogeneity have classically focused on abiotic heterogeneity^48^,^49^. Our study is the first to investigate clonal response to a heterogeneous distribution of AM fungi, based on the assumption that AM fungi can be regarded as a resource for the plant. However, in response to the heterogeneous distribution of AM fungi, *G. hederacea* clones displayed only a weak foraging response and no specialization which suggests, respectively, that clones do not aggregate more especially in patches with AM fungi or maximize their proportion of roots in contact with AM fungi. We provide a first explanation by highlighting the impact of AM fungal identity on the plant phenotypes and more particularly on the allocation traits involved in specialization. More importantly, we provide evidence that stolons might be vectors for the transfer of micro-organisms between ramets, thereby buffering (through this dispersion of fungi) the initial heterogeneous distribution. If this is true, stolons will have to be regarded in a different way, and be seen as ecological corridors for the dispersion of micro-organisms allowing a continuity of partnership along the clone. Considering the plant as a holobiont^31^,^50^, this novel view of stolon function is expected to stimulate new ideas and understanding about the heritability of microbiota in clonal plants.

## Methods

### Biological material

We used the clonal, perennial herb *Glechoma hederacea,* which is a common Lamiaceae in woods and grasslands. *G. hederacea* clones produce new erect shoots at the nodes at regular intervals of 5 to 10 cm on plagiotropic monopodial stolons (*i.e.* aboveground connections). Each ramet consists of a node with two leaves, a root system and two axillary buds. In climatic chambers with constant conditions, *G. hederacea* does not flower and displays only vegetative growth^12^. This species is known to exhibit foraging behavior^12^,^22^,^45^ and organ specialization^22^ in response to nutrients or light heterogeneity. The ramets used in our experiments were obtained from the vegetative multiplication of 10 clonal fragments taken in 10 different locations sufficiently spaced to obtain different genotypes. Plants were cultivated for three months under controlled conditions to avoid parental effects linked with their original habitats^51^. Vegetative multiplication was carried out on a sterilized substrate (50% sand and 50% vermiculite, autoclaved at 120 °C for 20 minutes) to ensure the absence of AM fungi propagules. For each experiment, the transplanted clonal unit consisted of a mature ramet (leaves and axillary buds) with one connective internode (to provide resources to support ramet survival)^52^, and without roots (to avoid prior mycorrhization). The AM fungi inocula used in both experiments were *Glomus* species: *Glomus intraradices* (see Stockinger *et al.*, 2009 for discussion on *G. intraradices* reclassification^53^), *Glomus custos*, and *Glomus clarum*. These AM species were chosen to limit phylogenetic differences between the fungal life-history traits^54^. G. intraradices has been shown to induce beneficial P uptake in *Medicago truncatula*^33^. The use of three different AM species also ensure a range of cooperativeness in the symbionts. The inocula used in the two experiments consisted of a single-species inoculum produced in *in vitro* root cultures (provided by S. L. Biotechnologia Ecologica, Granada, Spain) or a mixture of equal proportions of all three inocula. The inoculations consisted of an injection of 1 mL of inoculum directly above the roots, and were administered when the plants had root lengths of 0.5 to 1 cm.

### Experimental conditions

Experiment 1 was designed to test the foraging and specialization responses of *G. hederacea* to the heterogeneous distribution of AM fungi. Experiment 2 tested the effect of the species of AM fungus on the plant traits involved in these responses.

Both experiments were carried out with cultures grown on the same sterile substrate (50% sand, 50% vermiculite) in a climate-controlled chamber with a diurnal cycle of 12 h day /12 h night at 20 °C. Plants were watered with deionized water every two days to check for nutrient availability. Necessary nutrients were supplied by watering the plants every 10 days using a fertilizing Hoagland’s solution with strongly reduced phosphorus content to ensure ideal conditions for mycorrhization (*i.e.* phosphorus stress)^55^,^56^,^57^. At each watering, the volumes of deionized water and fertilizing solution per pot were 25 mL and 250 mL respectively for the first and second experiments. We also controlled nutrient accumulation during the experimental period by using pierced pots that allowed evacuation of the excess watering solution. To prevent nutrient enrichment due to the inoculum, AM fungi-free pots were also inoculated with a sterilized inoculum (autoclaved at 100 °C for five minutes).

### Experiment 1: Effect of heterogeneous AM fungal distribution on *G. hederacea* foraging and specialization responses

The responses of *G. hederacea* to four different spatial distributions of AM fungi were tested. *G. hederacea* was grown in series of 11 consecutive pots: two homogeneous treatments with the presence (P) or absence (A) of AM fungi in all pots; and two heterogeneous treatments with two patches of 5 pots either in presence then absence (PA) or absence then presence (AP) (Fig. 1). The two latter treatments were included to take into account a potential effect of ramet age in the plant’s response to heterogeneity. These treatments were replicated for 10 clones of Glechoma hederacea (see Methods section “Biological material” for precision on plants used). Each clone was grown in plastic pots (8 × 8 × 7 cm3) filled with sterile substrate. Only one ramet was allowed to root in each pot and plant growth was oriented in a line by removing lateral ramifications. The initial ramet, in all treatments, was planted in a pot without AM fungi. For each treatment, the inoculum consisted of a mixture of the three AM fungal species (*G. clarum*, *G. custos* and *G. intraradices*). Inoculations were started on the second pot of each line which actually contained the fourth ramet of the clone (exceptionally, the first three ramets rooted in the same first pot due to internode shortness, see Fig. 1). Inoculations were administered to each ramet separately when the ramet had roots 0.5 to 1 cm in length to avoid a ramet age effect on the AM fungi colonization process.

**Figure 6:**
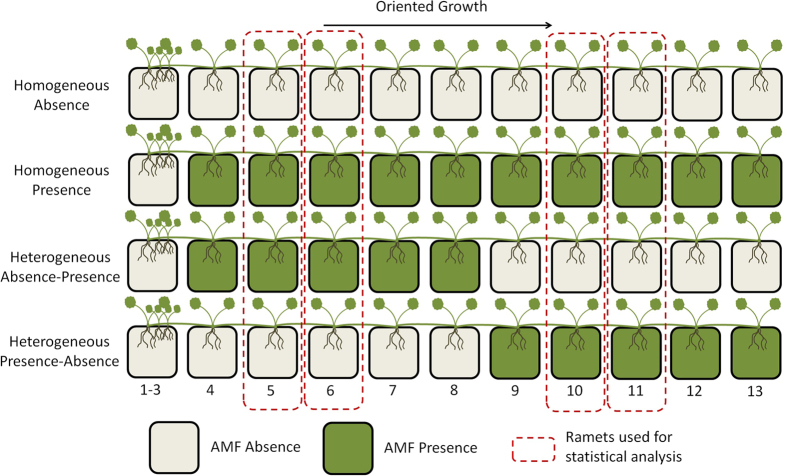
Schematic drawing of the experimental design composed of pots arranged in lines. Ramets were forced to root in different pots and lateral ramifications were removed to orient growth in a line. Four treatments of AM fungal distribution were applied based on the presence or absence of AM fungi in the pots: Absence (A) (10 pots without AM fungi); Presence (P) (10 pots with AM fungi); Presence-Absence (PA) (five pots with AM fungi followed by five pots without AM fungi); Absence-Presence (AP) (five pots without AM fungi followed by five pots with AM fungi).

The clones were harvested when the final ramet (number 13) had rooted in the 11th pot. This ensured that each clone had 10 points for sampling environmental quality. The 5th, 6th, 10th and 11th ramets of each clone in the pot line (Fig. 6) were used for statistical analyses. These ramets corresponded to the second and third ramets experiencing the current patch quality. Indeed, Louâpre *et al.* (2012) emphasized the role of the “past experience” of the clone in developing a plastic response. The choice of these four ramets thus ensured that the clone had enough sampling points to assess the quality of its habitat *i.e.* in the patches where AM fungi were present or absent, in the heterogeneous treatments, and to adjust accordingly when initiating new ramets^35^. Each ramet was carefully washed after harvesting. The foraging response was assessed by measuring the length of the internode just after the ramet. An aggregation of ramets, with shortened internodes, was expected in patches where AM fungi were present, and an avoidance of patches, *i.e.* production of longer internodes, was expected where AM fungi were absent. Modifications in ramification production linked to the effect of the treatment were checked by recording the number of ramifications produced by the ramets throughout the experiment. The specialization response was examined by measuring the root/shoot ratio (R/S) *i.e.* the biomass allocated to the below- and above-ground resource acquisition systems, after separating the roots and shoots and after oven-drying for 72 h at 65 °C. We expected a higher R/S ratio in patches where AM fungi were present than in patches where AM fungi were absent. Clone performance was assessed from (i) the total biomass of the clone, calculated as the sum of ramet roots, shoots and stolons after oven-drying for 72 h at 65 °C and (ii) the growth rate calculated as the number of days needed for the clone to develop the 10 sampling ramets *i.e.* the number of days between rooting of the 4th ramet and final harvesting.

### Experiment 2: Effect of AM fungal identity on *G. hederacea* performance and traits

The effects of individual AM fungal species on *G. hederacea* foraging and specialization traits were tested using four culture treatments: 1) no AM fungi, 2) with *Glomus custos*, 3) with *Glomus intraradices*, and 4) with *Glomus clarum*. Each treatment was replicated eight times with four related ramets assigned to each treatment replicate (32 clones in total), to control for plant-genotype effects. The initial ramet of each clone had previously been cultivated on sterile substrate to ensure root system development and facilitate survival after transplanting. The initial ramets were then transplanted in pots (27.5 × 12 × 35 cm3) filled with substrate. The AM fungi inoculations consisted of three injections of 1 mL of inoculum directly on the roots of the first three rooted ramets to ensure colonization of the whole pot. The plants were harvested after six weeks. The following traits involved in foraging were measured: (i) the longest primary stolon length (of order 1) as an indicator of the maximum spreading distance of space colonization (ii) the number of ramifications as an indicator of lateral spreading and clone densification. We also measured biomass allocation to the roots, shoots and stolons at the clone level, *i.e.* traits involved in the specialization response, after oven-drying for 72 h at 65 °C. Plant performance for the entire clone was determined from: (i) the total biomass calculated as the sum of the dry weights of the shoots, roots and stolons after oven-drying for 72 h at 65 °C. and (ii) the number of ramets *i.e.* the number of potential descendants. Performance was expected to be higher in pots inoculated with fungi and to differ depending on the fungal species.

### Statistical analysis

For experiment 1, to test whether *G. hederacea* developed a plastic foraging (internode length) or specialization (R/S ratio) response to the heterogeneous distribution of AM fungi, ANOVA analyses were performed using the linear mixed-effects model procedure in R 3.1.3^58^ with packages “nlme”^59^ and “car”^60^. Ramets of the same age were compared between genotypes to control for a possible effect of ramet age.

For experiment 2, to determine whether the species of AM fungi induced changes in plant traits and performance, ANOVA analyses were performed using linear mixed models with the same R packages and version described above. Resource allocation was tested by using the clone total biomass as covariate to take into account the trait variance associated with clone growth.

In both experiments genotype-induced variance and data dependency was controlled by considering the treatment (four modalities) as a fixed factor and the plant-clone genotype as a random factor. The effect of genotype was assessed by comparing the intra- and inter-genotype variance and was considered significant when the inter-genotype variance was strictly higher than the intra-genotype variance. When a significant effect of treatment was detected by ANOVA, post hoc contrast tests were performed using the “doBy” package^61^ to test for significant differences between modalities. When necessary, the normality of the residuals was checked by subjecting the data to log transformation. The total clone biomass (summed dry weights of shoots, roots, and stolons) was used as covariate to account for variance due to differences in clone performance.

## Additional Information

**How to cite this article**: Vannier, N. *et al.* AM fungi patchiness and the clonal growth of *Glechoma hederacea* in heterogeneous environments. *Sci. Rep.* **6**, 37852; doi: 10.1038/srep37852 (2016).

**Publisher's note:** Springer Nature remains neutral with regard to jurisdictional claims in published maps and institutional affiliations.

## Supplementary Material

Supplementary Information

## Figures and Tables

**Table 1:** Results of linear models for each trait linked to the plants foraging, specialization and performance.

Trait	Treatment		Total biomass		Random factor (Genotype)	
	F-value	P-value (α = 0.05)	F-value	P-value (α = 0.05)	Intra: lower/estimate/upper	Inter : lower/estimate/upper
Total Biomass	0.39	0.75	—	—	0.72/0.95/1.25	0.43/0.81/1.53
Growth time	2.7	0.067	—	—	3.98/5.24/6.89	0.37/1.79/8.53
5^th^ internode length	0.45	0.71	1.58	0.22	1.19/1.61/2.18	0.75/1.45/2.8
6^th^ internode length	1.92	0.15	**8.32**	**<0.01**	1.03/1.4/1.9	0.34/0.83/2.05
10^th^ internode length	**5.74**	**<0.01**	**4.38**	**<0.05**	0.59/0.81/1.12	0.54/0.97/1.74
11^th^ internode length	0.15	0.93	0.02	0.87	0.96/1.34/1.86	0.41/0.94/2.17
5^th^ ramet root/shoot	0.48	0.69	—	—	n/a	n/a
6^th^ ramet root/shoot	0.18	0.9	—	—	n/a	n/a
10^th^ ramet root/shoot	1.09	0.37	—	—	n/a	n/a
11^th^ ramet root/shoot	0.46	0.7	—	—	n/a	n/a
5^th^ ramet number of ramifications	1.1	0.36	**14.49**	**<0.01**	0.99/1.31/1.7	0.38/0.83/1.8
6^th^ ramet number of ramifications	0.46	0.7	**5.2**	**<0.01**	1.06/1.40/1.85	0.81/1.46/2.64
10^th^ ramet number of ramifications	0.26	0.84	1.91	0.18	0.89/1.18/1.55	0.36/0.77/1.66
11^th^ ramet number of ramifications	0.88	0.46	1.08	0.3	0.89/1.18/1.56	0.22/0.59/1.63

F-values and P-values of the treatment and total biomass (when used as covariable) are presented, as well as lower, estimate and upper values of intra and inter genotype variance (random factor).

**Table 2:** Results of linear models for each trait linked to the plants resources allocation and performance.

Trait	Treatment		Total biomass		Random factor (Genotype)	
	F-value	P-value (α = 0.05)	F-value	P-value (α = 0.05)	Intra: lower/estimate/upper	Inter : lower/estimate/upper
Total Biomass	0.67	0.57	—	—	0.27/0.38/0.52	0.03/0.14/0.67
Number of ramets (allocation)	**3.55**	**<0.05**	**46.6**	**<0,001**	5.97/8.45/11.96	7.58/13.7/24.8
Primary stolon length	2.84	0.07	1.99	0.17	10.99/15.45/21.75	4.53/10.69/25.23
Number of ramifications	1.49	0.25	**5.8**	**<0.05**	0.46/0.66/0.93	0.24/0.53/1.18
Stolons weight (allocation)	**4.51**	**<0.05**	**91.37**	**<0.001**	0.08/0.11/0.17	0.03/0.09/0.22
Shoots weight (allocation)	**3.96**	**<0.05**	**1528**	**<0.001**	0.06/0.09/0.13	0.04/0.08/0.18
Roots weight (allocation	0.5	0.68	**30.72**	**<0.001**	0.06/0.09/0.12	0.006/0.03/0.19

F-values and P-values of the treatment and total biomass (when used as covariable) are presented, as well as lower, estimated and upper values of intra and inter genotype variance (random factor).
