# Supplementary material for: AM fungi patchiness and the clonal growth of Glechoma hederacea in heterogeneous environments
Source: Sci Rep. 2016 Nov 25;6:37852. doi: 10.1038/srep37852 (PMC5122940; doi:10.1038/srep37852)
Supplement: Supplementary Information [file srep37852-s1.doc]

**AM fungi patchiness and the clonal growth of *Glechoma hederacea* in heterogeneous environments**

Nathan Vannier^1^, Anne-Kristel Bittebiere^2^, Philippe Vandenkoornhuyse^1^, Cendrine Mony^1^

address:

1 Université de Rennes 1, CNRS, UMR 6553 EcoBio, Campus Beaulieu, Avenue du Général Leclerc, 35042 RENNES Cedex (France)

2 Université de Lyon 1, CNRS, UMR 5023 LEHNA 43 Boulevard du 11 Novembre 1918, 69622 VILLEURBANNE Cedex (France)

**Supplementary informations**

**Imaging**

For scanning electron microscopy, samples were fixed with a fixative containing 4% glutaraldehyde in 0.,05M PHEM buffer (Pipes, EGTA and MgSO4) at RT. The samples were then rinsed and post-fixed for 1h at 4°C in buffered 1,% OsO4 buffered. The fFixed samples were dehydrated by passage through an ethanol series and finally dried using a critical point dryer (Baltec CPD 030, Balzer). The dried plant tissues were coated with gold in a sputter coater before being viewed with a PHENON electron microscope.

For transmission electron microscopy (TEM), samples were fixed for in a fixative containing 4% glutaraldehyde in 0.1 M sodium cacodylate buffer, pH 7.4. The samples were then rinsed in sodium cacodylate and post-fixed for 1h at 4 °C in 1% osmium tetroxide buffered in 0.1M of sodium cacodylate. Dehydration was carried out in a graded alcohol series (from 30 to 100%) and samples were finally embedded ing in Spurr. Sections were cut using a diamond knife on a Leica ultracut UCT ultramicrotome and, after staining with 2% uranyl acetate and 2% lead citrate, the grids were examined with a Jeol 1400 transmission electron microscope (Tokyo, Japan).
